# De-escalation of antimicrobial therapy in Gram-negative sepsis: easier said than done?

**DOI:** 10.1186/cc9639

**Published:** 2011-03-11

**Authors:** L Phee, N Gordon, M Hornsey, D Wareham

**Affiliations:** 1 Barts and The London NHS Trust, London, UK; 2 QMUL, London, UK

## Introduction

Appropriate and timely de-escalation of antimicrobial therapy has long been recognised as an important element in the optimal management of sepsis. When a causative pathogen has been isolated and its susceptibility profile known, the most suitable single therapy should be instituted to prevent the development of super-infection with pathogenic or resistant organisms, as well as reduce toxicity and costs.

## Methods

All blood cultures analysed on the BD BACTEC system were evaluated over a 6-week period (17 April 2010 to 30 May 2010). Organisms in positive blood cultures were then further identified by MALDI-TOF mass spectrometry (Brucker) and susceptibility testing was performed using the MicroScan WalkAway system (Siemens). The demographics, treatment regimens and clinical outcomes of all episodes of clinically significant Gram-negative bacteraemia were prospectively audited.

## Results

Two hundred and seventy sets of blood cultures were positive during our study period, representing 246 individual bacteraemic episodes. A total 143/270 were considered contaminants, 42/270 were significant Gram-positive bacteraemias, 1/270 *Candida albicans*, and 84/270 Gram-negative bacteraemia. Of the latter, 70 were individual episodes, of which two died prior to susceptibility results being available. Of the survivors, following knowledge of the susceptibility profile, only 20.5% (14) were de-escalated to a narrower-spectrum agent and only 31% (21) were converted to suitable oral agents when practical. Twelve per cent (10) were treated with combination therapies even though single agents remained highly active. Of the group that failed to de-escalate antimicrobial therapy (54 patients), the 30-day mortality rate was 9% (5/54) versus 7% (1/14) in the group (14 patients) that adhered to the surviving sepsis guidelines. Likewise, the former group were more likely to develop diarrhoea, 38% (21/54) versus 21% (3/14), with three patients positive for *Clostridium difficile *toxin in the former group (none in the latter). Multidrug-resistant organisms and fungal colonisation occurred more frequently in the first group - 38% (21/54) and 22% (12/54) versus 21% (3/14) and 0% (0/14), respectively.

## Conclusions

Surviving sepsis guidelines have reiterated the need for timely use of appropriate empirical antimicrobials as well as the importance of de-escalation of therapy when the causative agent has been identified. However, as is the case in our study, this has not always been the prevailing practice. Many factors underlie this deviation from recommended guidelines, including worsening clinical condition, reported penicillin allergy as well as multiple co-morbidities.

